# Detection and assessment of immune and stromal related risk genes to predict preeclampsia: A bioinformatics analysis with dataset

**DOI:** 10.1097/MD.0000000000038638

**Published:** 2024-06-28

**Authors:** Hong Qin

**Affiliations:** a Obstetrics Department, Longhua District Maternal and Child Health Care Hospital, Shenzhen, China.

**Keywords:** diagnostic biomarker, immune score, placenta, preeclampsia, WGCNA

## Abstract

This study aimed to investigate immune score and stromal score-related signatures associated with preeclampsia (PE) and identify key genes for diagnosing PE using bioinformatics analysis. Four microarray datasets, GSE75010, GSE25906, GSE44711, and GSE10588 were obtained from the Gene Expression Omnibus database. GSE75010 was utilized for differential expressed gene (DEGs) analysis. Subsequently, bioinformatic tools such as gene ontology, Kyoto Encyclopedia of Genes and Genomes, weighted gene correlation network analysis, and gene set enrichment analysis were employed to functionally characterize candidate target genes involved in the pathogenesis of PE. The least absolute shrinkage and selection operator regression approach was employed to identify crucial genes and develop a predictive model. This method also facilitated the creation of receiver operating characteristic (ROC) curves, enabling the evaluation of the model’s precision. Furthermore, the model underwent external validation through the other three datasets. A total of 3286 DEGs were identified between normal and PE tissues. Gene ontology and Kyoto Encyclopedia of Genes and Genomes analyses revealed enrichments in functions related to cell chemotaxis, cytokine binding, and cytokine–cytokine receptor interaction. weighted gene correlation network analysis identified 2 color modules strongly correlated with immune and stromal scores. After intersecting DEGs with immune and stromal-related genes, 13 genes were selected and added to the least absolute shrinkage and selection operator regression. Ultimately, 7 genes were screened out to establish the risk model for discriminating preeclampsia from controls, with each gene having an area under the ROC curve >0.70. The constructed risk model demonstrated that the area under the ROC curves in internal and the other three external datasets were all greater than 0.80. A 7-gene risk signature was identified to build a potential diagnostic model and performed well in the external validation group for PE patients. These findings illustrated that immune and stromal cells played essential roles in PE during its progression.

## 1. Introduction

Preeclampsia (PE) is a medical condition that occurs exclusively during pregnancy. It manifests as elevated blood pressure and the presence of protein in the urine (proteinuria), typically after the 20-week gestation mark in women who previously had normal blood pressure readings.^[1]^ It affects up to 5% of pregnant women globally and presents with diverse clinical subtypes, making it one of the leading contributors to maternal and fetal morbidity and mortality during pregnancy.^[2]^ Currently, the options for clinically managing preeclampsia are relatively few and generally revolve around the delivery of the baby. Moreover, even postpartum, there is a substantial risk that both mothers and newborns may face long-term health issues, such as chronic high blood pressure or kidney diseases.^[3]^ Hence, it is of utmost importance to promptly identify pregnant women at a high risk of developing PE or severe PE and to discover novel drug therapy targets. These measures are crucial for alleviating adverse pregnancy outcomes. Substantial advancements have been made in understanding how environmental stimuli and genetic factors impact the onset of PE. However, the precise etiology and pathophysiology of the disease remain elusive, necessitating thorough investigation. The pivotal involvement of the placenta in the development of the disease is widely acknowledged, underscoring the significance of studying early-onset PE for insights into placental pathophysiological changes.^[4,5]^

Many diseases exhibit systemic development and are influenced by more than just individual genes. In the context of PE, placental insufficiency may arise from the intricate interplay of a cluster of specific genes, contributing to its complexity.^[6]^ Consequently, we posited that exploring the key genes contributing to PE through cooperative gene modules was crucial. In recent decades, investigators have focused extensively on identifying transcriptomic alterations in placental tissues of women with PE using microarray technology and bioinformatic analysis. These efforts have enabled the identification of differential expressed gene (DEGs) involved in the pathogenesis of PE.^[7]^ There have been several gene expression profile studies about PE on Gene Expression Omnibus (GEO) database.^[8]^ In a particular study, DEGs associated with cell–cell or cell-extracellular matrix interaction (ITGA5, SPP1, LUM, VCAN, APP), as well as placental metabolic or oxidative stress (CCR7, NT5E, CYBB), were identified as potentially crucial genes. These genes were predicted to play significant roles in the pathogenesis of early-onset PE.^[9]^ Weighted gene correlation network analysis (WGCNA) is a tool that focuses on genome-wide expression.^[10]^ WGCNA is utilized to identify potential gene modules linked to disease traits by investigating the influence and regulatory connections among genes that share similar expression patterns.

In this study, to investigate potential crucial genes in PE compared with gestational age matched controls, the microarray data deposited by Liang M^[11]^ were used to identify DEGs. gene ontology (GO), Kyoto Encyclopedia of Genes and Genomes (KEGG) pathway enrichment analysis, and WGCNA analysis were further performed to help us understand the molecular mechanisms underlying the pathogenesis of PE. LASSO is applied to uncover potential gene modules associated with disease traits by investigating the influence and regulatory connections among genes exhibiting comparable expression patterns.

## 2. Materials and methods

### 2.1. Data collection and preprocessing

We obtained 4 mRNA datasets, GSE75010, GSE25906,GSE44711, and GSE10588 from the GEO database (https://www.ncbi.nlm.nih.gov/geo/).^[12,13]^ The GSE75010 dataset consisted of 157 placenta samples, including 80 samples from preeclampsia patients and 77 samples from control patients. The GSE75010 dataset served as the training dataset, while the GSE25906, GSE44711, and GSE10588 datasets were utilized as the external validation dataset. Initially, we transformed the probe numbers in all datasets into gene symbols and removed any null probes using the R language. Afterward, all datasets were normalized using the Robust Multi-Array Average method and log_2_ transformed using the R language. Furthermore, the ESTIMATE algorithm from the downloaded database was employed to evaluate the overall stromal content (Stromal Score), immune infiltration (Immune Score), and the combined score (ESTIMATE Score) for each sample.^[14]^ Because all data for this study were obtained from public databases, the study did not require the institutional review board approval.

### 2.2. Identification of DEGs

Limma package was used in R language to perform the differential expression analysis between preeclampsia samples and control samples of GSE75010 datasets.^[15]^ The difference of immune scores and stromal scores among patients in different immune risk groups was expressed by “ggplot2” and “ggsignif ” (R package).^[16]^ Differential expressed genes (DEGs) were considered as significant when the |fold change (FC)| > 1.0 and adjusted *P*-value < 0.05. The visualization of these genes was plotted using “pheatmap” and “ggpuber” package in R.

### 2.3. Functional and pathway enrichment analysis of DEGs

To explore the possible functions of DEGs, GO and KEGG pathway enrichment analysis were performed using “clusterProfiler” in R language.^[17,18]^ *P* value < .05 was deemed as significant. The results of these analysis were plotted via “ggplot2” package in R.

### 2.4. WGCNA

WGCNA was performed using the “WGCNA” package in R software to construct and visualize the network, exploring potential interactions among genes.^[10]^ Initially, we calculated sample similarities through horizontal hierarchical clustering. Next, we constructed a weighted adjacency matrix and determined an appropriate soft threshold, ensuring a high correlation and average connectivity to achieve a scale-free model. A cluster dendrogram was created to visualize modules represented by different colors, and dynamic modules with high similarities were combined into one at the cutline. Subsequently, we calculated the correlation between the stromal/immune score and each gene module to identify modules strongly associated with the score. Genes from the significant modules were selected as subtype-related hub genes for further analysis.

### 2.5. Construction of logistic regression model and diagnostic role of hub genes in PE

The least absolute shrinkage and selection operator (LASSO) method was performed using “glmnet” package^[19]^ in R language to screen out genes constructing logistic regression model. Next, the genes were employed to construct a logistic regression model in GSE75010. To further investigate the diagnostic significance of the hub genes in PE, we conducted receiver operating characteristic (ROC) experiments, and their diagnostic potential was assessed by calculating the area under the ROC curve (AUC). Genes with an AUC > 0.7 and a *P*-value < 0.05 were regarded as potential diagnostic markers. Additionally, we utilized the “ROC” package to plot the ROC curve, evaluating the reliability of the logistic regression model.^[20]^ Furthermore, the GSE25906, GSE44711, and GSE10588 datasets were used as the external validation datasets.

### 2.6. Immunohistochemistry

A total of 6 normal placenta tissues and 6 PE tissues were collected for immunohistochemistry (IHC) analysis. The collection of these tissues was approved by the Institutional Review Boards of Longhua District Maternal and Child Health Care Hospital. Comprehensive demographic information was gathered from the electronic records. The IHC analysis for COL17A1, BHLHE40, FSTL3, and SH3BP5 in both PE and normal placenta tissues (control group) was carried out following previously described methods.^[21]^ The Image-pro Plus 6.0 was used to assess the expression level of COL17A1, BHLHE40, FSTL3, and SH3BP5 by integrated optical density (IOD) (integrated optical density) value.

### 2.7. Statistical analysis

Continuous variables were summarized as mean ± SD ; categorized variables were described by number (n) and proportion (%). Differences among variables were tested using t-tests, nonparametric tests, chi-square tests, or ANOVA tests. *P*-value <.05 was considered statistically significant.

## 3. Results

### 3.1. Distribution of immune and stromal infiltration in different features of PE patients

**Figure 1.**
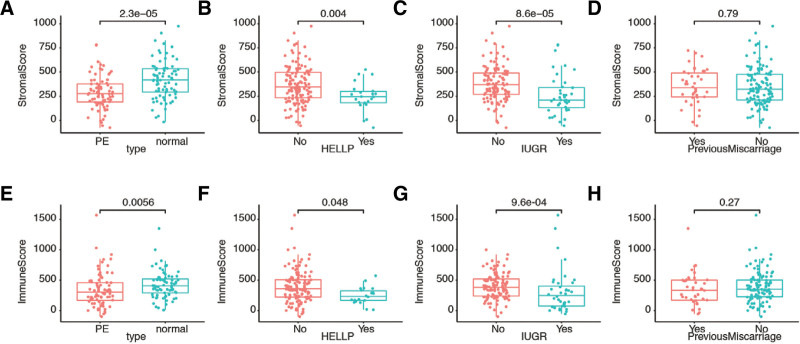
Distribution of stromal score and immune score in different clinicopathological characteristics. Stromal score in different (A) placenta tissues (*P* = 2.3e−05). (B) HELLP status (*P* = .004). (C) IUGR status (*P* = 8.6e−05). (D) previous miscarriage (*P* = .79). Immune score in different (E) placenta tissues (*P* = .0056). (F) HELLP status (*P* = .048). (G) IUGR status (*P* = 9.6e‐04). (H) previous miscarriage (*P* = .27). HELLP = hemolysis, elevated liver function and low platelet count syndrome, IUGR = intrauterine growth retardation.

We calculated stromal and immune scores for each patient and compared the scores among different clinicopathological characteristics. The stromal scores were found to be higher in the normal subgroup (Fig. 1A), in patients without hemolysis, elevated liver function and low platelet count syndrome (Fig. 1B), and in patients without intrauterine growth retardation (Fig. 1C). However, no significant differences were observed between patients with or without a history of previous miscarriage (Fig. 1D). Conversely, the distribution of immune scores displayed similar patterns among patients with different features, including PE type (Fig. 1E), presence or absence of hemolysis, elevated liver function and low platelet count syndrome (Fig. 1F), presence or absence of intrauterine growth retardation (Fig. 1G), and whether they had experienced a previous miscarriage or not (Fig. 1H). Overall, these results indicate that both stromal scores and immune scores significantly differ among patients with PE.

### 3.2. Identification of DEGs with different stromal and immune score

**Figure 2.**
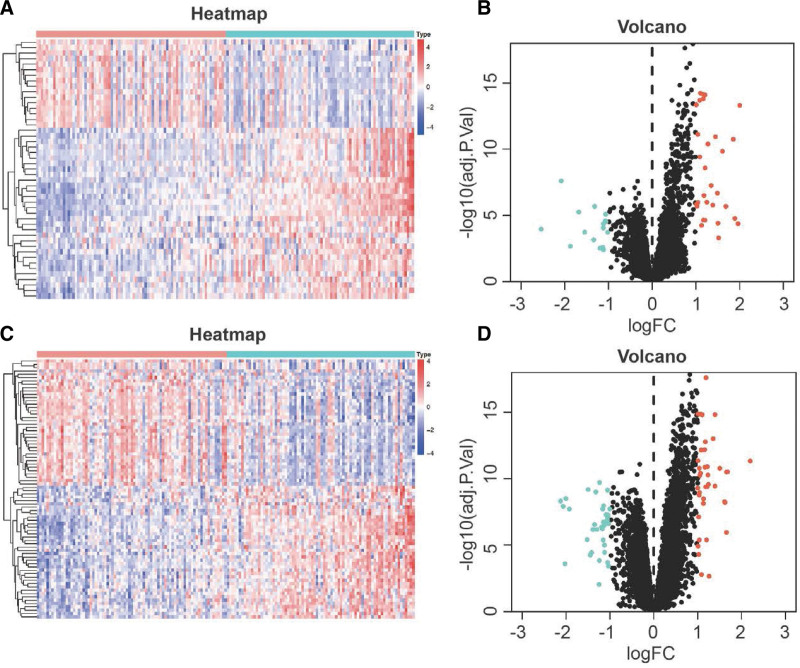
Differentially expressed genes (DEGs) between PE patients with low and high immune score or stromal score. (A) Heatmap and (B) volcano plots between high and low immune scores in PE patients. (C) Heatmap and (D) volcano plots between high and low stromal scores in PE patients. PE = preeclampsia.

Next, we investigated the differentially expressed genes between patients with low and high stromal or immune score groups in the GSE75010 dataset. From this analysis, we identified a total of 47 differentially expressed genes, with 31 being up-regulated and 16 down-regulated, based on the comparison of low and high immune scores (Fig. 2A and B). Similarly, applying the same criterion, we obtained 78 differentially expressed genes between high stromal infiltration and low stromal infiltration clusters in the database, with 40 genes being up-regulated and 38 down-regulated (Fig. 2C and D). In total, we obtained 125 DEGs, consisting of 71 up-regulated genes and 54 down-regulated genes. These genes were subjected to further analysis in the subsequent study.

### 3.3. Functional enrichment analysis of DEGs

**Figure 3.**
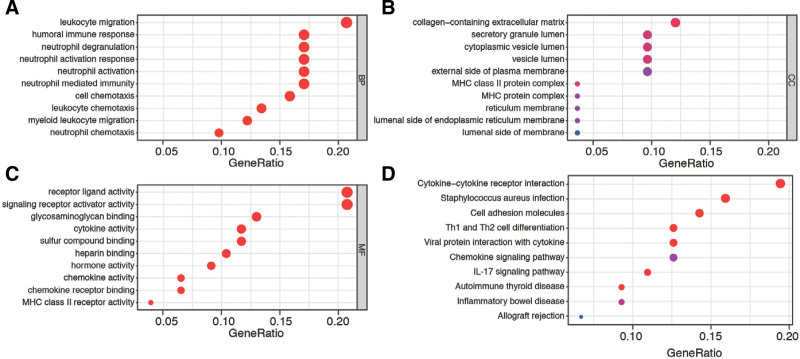
Enrichment and functional analysis of DEGs between normal and PE placenta tissues. (A) The top 10 biological process terms of GO analysis. (B) The top 10 cell component terms of GO analysis. (C) The top 10 molecular function terms of GO analysis. (D) The top 10 most enriched KEGG pathways of DEGs. DEGs = differential expressed gene, GO = gene ontology, PE = preeclampsia.

In order to gain insight into the biological functions of gene sets involved in stromal and immune-related DEGs, we conducted GO enrichment and KEGG pathway analyses. The GO results revealed significant enrichment of DEGs in crucial biological functions, including leukocyte migration, collagen-containing extracellular matrix, and receptor ligand activity, all of which are closely correlated with the pathogenesis of placental insufficiency in PE (Fig. 3A–C). Furthermore, the KEGG pathway analysis demonstrated that these DEGs are enriched in pathways such as cytokine–cytokine receptor interaction, cell adhesion molecules, and Th1 and Th2 cell differentiation (Fig. 3D). These functions and pathways may be crucial in the development and advancement of PE.

### 3.4. Gene co-expression modules correspond to clinical traits

**Figure 4.**
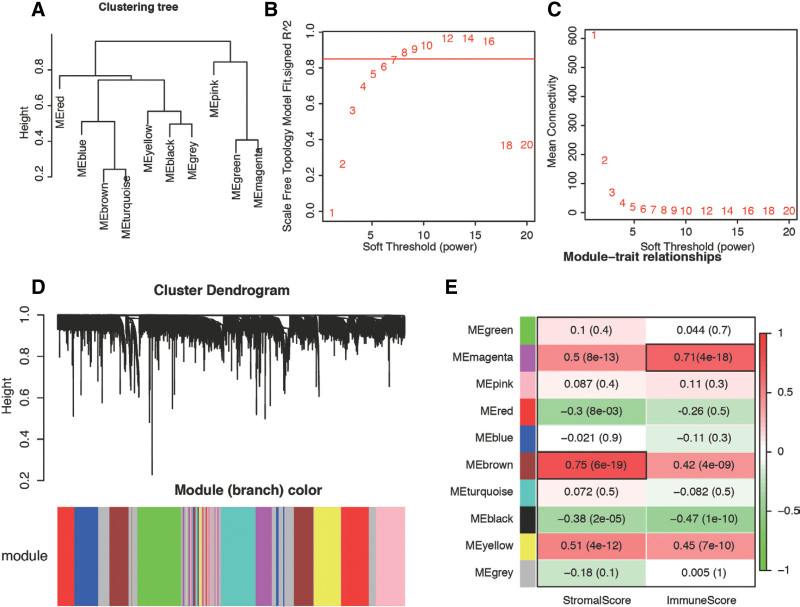
Determination of soft-thresholding power in WGCNA. (A) The eigengene dendrogram and heatmap identify groups of correlated eigengenes termed meta-modules. (B) Mean connectivity analysis of various soft-thresholding powers. (C) Histogram of the connection distribution when β = 7. (D) Clustering dendrograms of genes based on dissimilarity topological overlap and module colors. (E) Correlation between modules and different immune traits, including stromal and immune score. WGCNA = weighted gene correlation network analysis.

We employed WGCNA to identify gene modules that exhibited correlations with the stromal score and immune score based on the DEGs between normal and PE tissues. The process of network constructions and module detections was conducted step-by-step. Initially, we visualized the gene network with meta-modules (Fig. 4A). The scale independence and mean connectivity with various soft thresholds (β) ranging from 1 to 20 are depicted in Figure 4B and C, respectively. At β = 7, the co-expression network distribution demonstrated an approximate scale-free topology. Furthermore, an eigengene dendrogram and heatmap were constructed to examine groups of correlated eigengenes and the dendrogram of all modules (Fig. 4D). In total, we obtained ten cohesive modules containing highly correlated DEGs. Subsequently, we investigated the association of these modules with sample traits, such as stromal score and immune score, which represented their infiltrating levels. As shown in Fig. 4E, the brown module exhibited the most significant correlation with the stromal score (cor = 0.75, *P* = 6e‐19), while the magenta module was significantly correlated with the immune score (cor = 0.71, *P* = 4e‐18). These 2 modules were then selected for the identification of highly connected intramodular genes (hub genes).

### 3.5. Construction and validation of the diagnostic genes signature

We employed WGCNA to identify gene modules that exhibited correlations with the stromal score and immune score based on the DEGs between normal and PE tissues. The process of network constructions and module detections was conducted step-by-step. Initially, we visualized the gene network with meta-modules (Fig. 4A). The scale independence and mean connectivity with various soft thresholds (β) ranging from 1 to 20 are depicted in Figure 4B and C, respectively. At β = 7, the co-expression network distribution demonstrated an approximate scale-free topology. Furthermore, an eigengene dendrogram and heatmap were constructed to examine groups of correlated eigengenes and the dendrogram of all modules (Fig. 4D). In total, we obtained ten cohesive modules containing highly correlated DEGs. Subsequently, we investigated the association of these modules with sample traits, such as stromal score and immune score, which represented their infiltrating levels. As shown in Fig. 4E, the brown module exhibited the most significant correlation with the stromal score (cor = 0.75, *P* = 6e‐19), while the magenta module was significantly correlated with the immune score (cor = 0.71, *P* = 4e‐18). These 2 modules were then selected for the identification of highly connected intramodular genes (hub genes).

### 3.6. Construction and validation of the diagnostic genes signature

**Figure 5.**
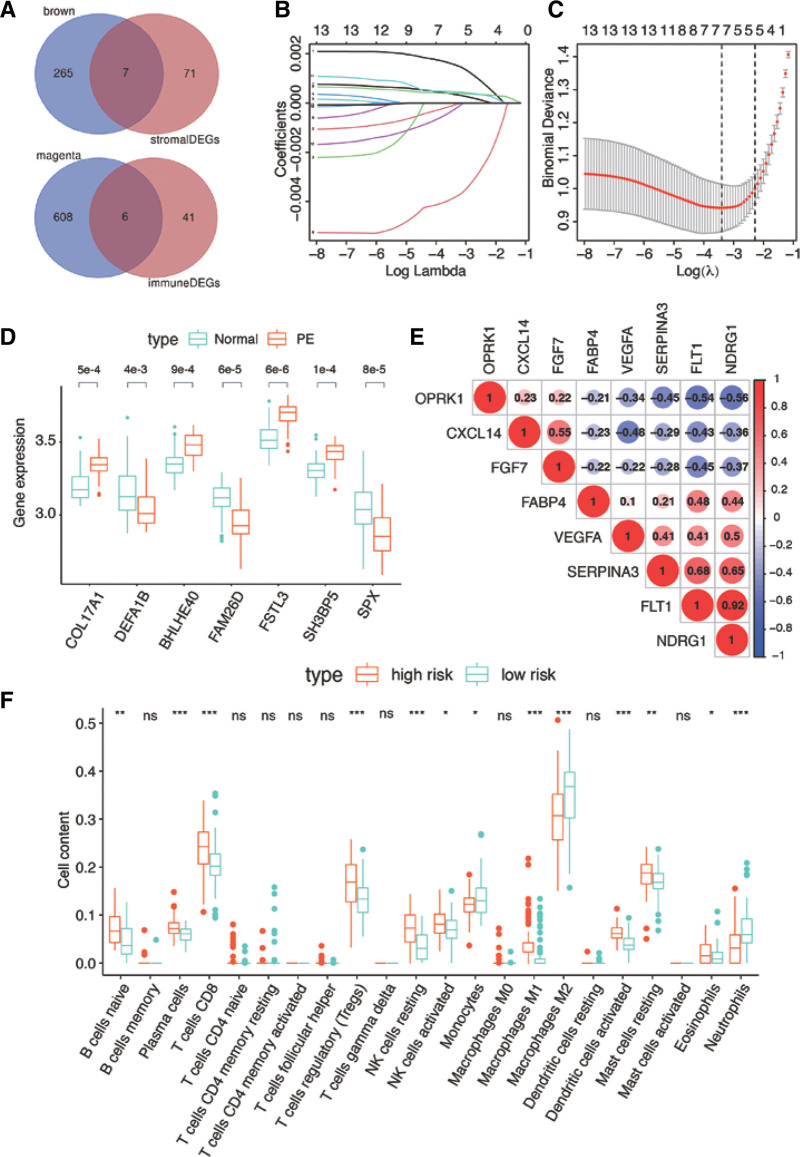
Identification and construction of risk signature for prediction of PE. (A) Venn diagram shows that there are 7 genes overlapping between brown module and stromal related DEGs, and 6 genes overlapping between magenta module and immune related DEGs. (B) Log (Lambda) value of the 13 genes in LASSO model. (C) Cross-validation for tuning parameter selection in the proportional hazards model, dotted vertical lines were drawn at the optimal values, and 7 genes were identified by LASSO regression. (D) Expression of 7 selected genes between normal and PE placenta tissues, and the 7 genes are all differentially expressed including COL17A1(*P* = 5.1e‐04), DEFA1B(*P* = 4.7e‐03), BHLHE40(*P* = 9.4e‐04), FAM26D(*P* = 6.9e‐05), FSTL3(*P* = 6.2e‐06), SH3BP5(*P* = 1.9e‐04), and SPX(*P* = 8.5e‐05). (E) Spearman correlation analysis of 7 selected genes. (F) The association of immune cells infiltration and the immune related risk signature in PE patients. *P < .05; **P < .01; ***P < .001; DEGs = differential expressed gene, LASSO = least absolute shrinkage and selection operator, ns = not significant, PE = preeclampsia.

Finally, through using Venn analysis between stromal related DEGs and brown module, 7 hub genes are acquired. Similarly, 6 hub genes are resulted from immune-related DEGs and magenta module. In total, 13 genes are identified in both groups (Fig. 5A). Then the LASSO regression model was employed to screen for the most robust biomarkers to create an infiltrating-related diagnostic signature in the dataset GSE75010 (Fig. 5B–C). Seven genes were identified to construct the diagnostic signature including COL17A1, DEFA1B, BHLHE40, FAM26D, FSTL3, SH3BP5, and SPX. The expressions of the 7 genes are compared between patients with or without PE. The results suggested that the contents of COL17A1 (*P* = 5.1e‐04), BHLHE40 (*P* = 9.4e‐04), FSTL3 (*P* = 6.2e‐06), and SH3BP5 (*P* = 1.9e‐04) are higher in PE groups. In addition, the expressions of DEFA1B (*P* = 4.7e‐03), FAM26D (*P* = 6.8e‐05), and SPX (*P* = 8.5e‐05) are low in PE groups (Fig. 5D). Moreover, we analyzed the interactions between the 7 hub genes that were identified as affecting patient prognosis in the model (Fig. 5E). We found that the relationship between FLT1 and NDRG1, FLT1 and SERPINA3, NDRG1 and SERPINA3 are significantly correlative.

**Figure 6.**
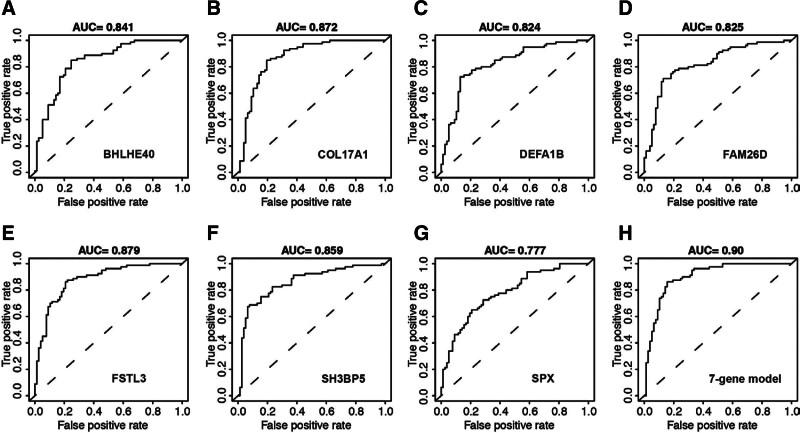
ROC curves show the predictive accuracy of PE for 7 different genes and the established risk signature. (A) AUC = 0.841 for BHLHE40. (B) AUC = 0.872 for COL17A1. (C) AUC = 0.824 for DEFA1B. (D) AUC = 0.825 for FAM26D. (E) AUC = 0.879 for FSTL3. (F) AUC = 0.859 for SH3BP5. (G) AUC = 0.777 for SPX. (H) AUC = 0.90 for 7-gene risk signature. AUC = area under the ROC curve, PE = preeclampsia. ROC = receiver operating characteristic.

Furthermore, risk scores are calculated by coefficient of each gene from LASSO regression multiple expression of each gene. The risk score formulation for this risk signature was established as follows: risk score = (0.0682 * COL17A1) ‐ (0.0546 * DEFA1B) + (0.0715 * BHLHE40) ‐ (0.0291 * FAM26D) + (0.1002 * FSTL3) + (0.1016 * SH3BP5) ‐ (0.0016 * SPX). Then, each patient was clustered into high- and low-risk groups according to the median value of risk score. CIBERSORT was applied to assess the relative proportion of the 22 immune cells in each placenta sample. As shown in Figure 5F, among the 22 immune cell types, B cells naïve, plasma cells, CD8+ T cells, Tregs, resting T cells, M1 macrophages, and neutrophils were positively correlated with the 7-gene risk model. To confirm the accuracy of the 7 genes and the risk model, we plot the ROC curves of the model in the dataset. The AUC values for predicting PE are outperformed for all of the 7 genes, including BHLHE40 (Fig. 6A, AUC = 0.841), COL17A1 (Fig. 6B, AUC = 0.872), DEFA1B (Fig. 6C, AUC = 0.824), FAM26D (Fig. 6D, AUC = 0.825), FSTL3 (Fig. 6E, AUC = 0.879), SH3BP5 (Fig. 6F, AUC = 0.859), SPX (Fig. 6G, AUC = 0.777). The accuracy of the risk model combining the 7 genes are significantly in predicting PE results (Fig. 6H, AUC = 0.900).

### 3.7. External validation with GSE25906 and IHC

**Figure 7.**
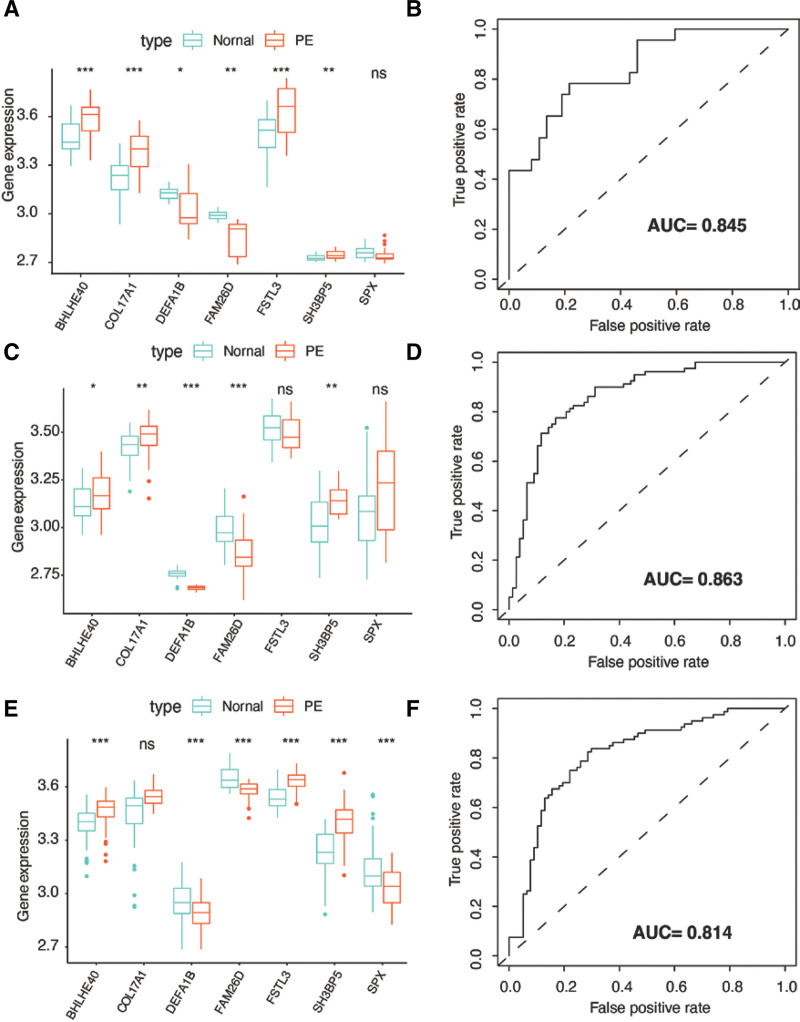
External validation for the 7-gene risk signature with other GEO datasets. (A) Expression of 7 genes between normal and PE placenta tissues in GSE25906. (B) ROC curve used for evaluating predictive accuracy of the risk signature in GSE25906 (AUC = 0.845). (C) Expression of 7 genes between normal and PE placenta tissues in GSE44711. (D) ROC curve used for evaluating predictive accuracy of the risk signature in GSE44711 (AUC = 0.863). (E) Expression of 7 genes between normal and PE placenta tissues in GSE10588. (F) ROC curve used for evaluating predictive accuracy of the risk signature in GSE10588 (AUC = 0.814). *P < .05; **P < .01; ***P < .001. GEO = Gene Expression Omnibus, PE = preeclampsia, ROC = receiver operating characteristic.

We confirmed the expression patterns of the 7 key genes using data from the GSE25906, GSE44711, and GSE10588 datasets. As shown in Fig. 7, the results confirmed that expressions of COL17A1, BHLHE40, FSTL3, and SH3BP5 were elevated in PE groups, while the expressions of DEFA1B and FAM26D were lower in PE groups in GSE25906 (Fig. 7A), GSE44711 (Fig. 7C), and GSE10588 (Fig. 7E). Subsequently, ROC analyses demonstrated that the AUC of the constructed risk model was also higher in GSE25906 (AUC = 0.845, Fig. 7B), GSE44711(AUC = 0.863, Fig. 7D), and GSE44711(AUC = 0.814, Fig. 7F). These results indicated that the predictive model had a relatively high diagnostic value in external datasets. Additionally, we use 7 genes in GSE25906 as training set to construct a risk model by LASSO regression and GSE75010 as validation set. As shown in Figure S1, Supplemental Digital Content, http://links.lww.com/MD/N8, similar results were obtained. AUC of the predictive model in GSE75010 is 0.872. We conducted IHC to detect the expression levels of 4 up-regulated genes in PE, namely COL17A1, BHLHE40, FSTL3, and SH3BP5, in 6 PE placenta tissues and 6 normal placenta tissues (control group). In Figure S2A, Supplemental Digital Content, http://links.lww.com/MD/N9, positive expression of the markers was indicated by the colors yellow and brown. The staining revealed that the four genes were expressed in both the cytoplasm and membrane, and visual inspection suggested higher expression rates in the PE group compared to the control group. To quantitatively analyze this effect, we assessed the IOD of the images and observed that the IOD was significantly higher in the PE group than in the control group (Fig. S2B, Supplemental Digital Content, http://links.lww.com/MD/N9). In conclusion, our risk model for predicting PE was successfully validated and exhibited superior performance when applied to external datasets and placental tissue.

## 4. Discussion

Preeclampsia (PE), a hypertensive disorder manifesting during pregnancy, remains a significant contributor to maternal and perinatal mortality and morbidity globally. The consequences of PE, such as fetal growth restriction, preterm birth, and various related complications, impose significant economic and psychological burdens on both affected families and society as a whole.^[22]^ Although the etiology of preeclampsia remains largely unclear, the main hypotheses strongly rely on disturbed placental function pregnancy. Placenta is the key organ involved in preeclampsia,^[23]^ and the identification of differentially expressed genes between normotensive and preeclamptic placentas is crucial to understand the molecular mechanism of the disease, contributing to the development of efficient biomarkers or therapeutic targets.^[24]^ Maternal immune tolerance, integral to the development of PE, is closely intertwined with the remodeling of spiral arteries and the infiltration of placental immune cells. Growing evidence suggests that preeclampsia constitutes a severe gestational disorder characterized by pronounced inflammation within the maternal body, disrupting the functionality of the monocyte–macrophage system.^[25]^ Immune cell infiltration analysis is a cutting-edge bioinformatic technique used to explore the diagnosis and prognosis of various diseases, including bladder,^[26]^ breast,^[27]^ colorectal,^[28]^ and ovarian.^[29]^ While numerous studies have diligently pursued molecular markers for diagnosing and treating PE through data exploration and analysis in databases like GEO, exploration of the immune and stromal cell infiltration landscape in PE and the molecular typing of immunomarkers remains unexplored.

In this study, we first calculated the stromal and immune score of each patient in GSE75010 dataset, and compared these scores in different clinicopathological characteristics. Then we analyzed the gene expression profiles of placenta samples between different immune related scores, and 125 DEGs were identified, suggesting that these differentially expressed genes might be potentially associated with the immune or stromal infiltration of PE during pregnancy. By employing WGCNA to create a weighted co-expression network of these genes, 2 co-expression modules were identified. Further analysis of the genes within the most pertinent brown and magenta modules, along with the previously mentioned DEGs, revealed 7 hub genes, including COL17A1 (collagen type XVII alpha 1 chain), DEFA1B (defensin alpha 1B), BHLHE40 (basic helix-loop-helix family member e40), FAM26D (family with sequence similarity 26 member D), FSTL3 (follistatin like 3), SH3BP5 (SH3 domain binding protein 5), and SPX (spexin hormone). Afterwards, a risk model based on the 7 genes was constructed to predict PE and immune or stromal infiltrating status in placenta samples. As a result, internal and external validation proved that the accuracy of the 7 genes and the integrated model was high for prediction. The AUC for indicating PE in GSE75010 was 0.900, and 0.845 for GSE25906. Our study contributes to unraveling the mechanisms behind PE and immune infiltration, with a particular emphasis on investigating the immune microenvironment within the placenta associated with PE. The 7 key genes we have identified hold promise as targeted candidates for the prevention and treatment of PE. Moreover, the predictive model for PE, which incorporates these 7 genes along with other indices, can enhance clinical practitioners’ ability to assess the prognosis of PE patients. Notably, a prior study also recognized a 7-gene risk model as a novel diagnostic biomarker for PE patients. This model concentrated on the HIF1-signaling pathway for discrimination PE from controls, of which the AUC are 0.923 and 0.845 in training and validation datasets respectively.^[30]^ Another study used bioinformatics to analyze DEmRNAs, DEmiRNAs, DEcircRNAs to speculate a competing endogenous RNA regulatory network in PE.^[31]^ Meng et al analyzed immune cell infiltration landscape in PE and found 41 hub genes that may be closely related to the molecular typing of PE. This study indicates that hub genes could be employed for categorizing PE into various subtypes.^[32]^ PE is also related to inflammatory response-related genes, 3 key inflammation-associated genes were identified and could be used as potential genetic biomarkers for preeclampsia prediction and treatment. Regulatory T cells (Tregs) and resting mast cells were found to be differentially distributed in 2 groups.^[33]^ Immune cell infiltration plays an essential role in early diagnosis and treatment for tumors. A predictive model was constructed by GOLT1B in TCGA dataset. The study provided a comprehensive understanding of the oncogenic role of GOLT1B, highlighting a potential mechanism whereby GOLT1B influences the tumor microenvironment, as well as cancer immunotherapy.^[34]^

Significant evidence suggests that immune and stromal infiltration play pivotal roles in the onset and progression of PE. A study reported the establishment of a 12-gene risk signature, which demonstrated high accuracy in predicting PE during pregnancy (AUC = 0.90).^[35]^ Wang et al suggested that LEP overexpression is associated with PE and may be a potential diagnostic marker and therapeutic target. Immune cell infiltration analysis showed that M1 and M2 macrophages differed between normal pregnancies and those in PE patients.^[36]^ Moreover, it has been reported that three inflammation-associated genes, namely INHBA, OPRK1, and TPBG, could serve as potential genetic biomarkers for predicting and treating PE. Furthermore, a nomogram has been developed as a predictive model to delineate the molecular mechanisms underlying preeclampsia pathogenesis and to predict drug responses at the transcriptome level.^[33]^ Additionally, immune cell infiltration in PE and normal samples was analyzed using CIBERSORT. We found significantly different infiltration between high and low risk groups of thirteen types of immune cell types. The literature indicates that decidual NK cells promote trophoblast invasion through chemokine secretion, while decidual macrophages function as antigen-presenting phagocytes, secreting cytokines and maintaining immune balance between mother and fetus.^[37]^ T cells and dendritic cells are recognized as pivotal cells in maintaining immune equilibrium.^[38]^ These reports affirm the significance of immune cell infiltration in the pathogenesis and classification of PE.

Moreover, 7 genes were screened out with LASSO to construct the regression model including COL17A1, DEFA1B, BHLHE40, FAM26D, FSTL3, SH3BP5, and SPX, which were found that significantly diversely expressed in different types of placentas, while its function in preeclampsia remained to be investigated. PA previous study identified COL17A1 and FSTL3 as novel diagnostic biomarkers through integrated bioinformatics analysis. Furthermore, exposure of primary human trophoblast cells to hypoxia-mimetic cobalt chloride resulted in upregulation of FSTL3 transcript expression.^[39]^ The downregulated FSTL3 remarkably inhibited the proliferative, migratory, and invasive abilities and lipid storage whereas elevated the programmed cell death of trophoblasts. Aberrant expression of FSTL3 in preeclampsia led to the dysfunction of trophoblast, indicating its involvement in the pathogenesis of preeclampsia.^[40]^ Increased FSTL-3 levels were linked to preeclampsia and correlated with a higher risk of subsequent preeclampsia development. The aberrant expression of FSTL3 in the condition disrupted trophoblast function, indicating its involvement in the disease’s etiopathogenesis. This mechanism involves enhancing placental lipid storage in PE patients.^[41]^ However, the expression and mechanisms of COL17A1 and SH3BP5 have not been investigated. BHLHE40 is a transcriptional repressor in response to hypoxia. Bioinformatics analysis demonstrated that BHLHE40 negatively regulates miR-196a-5p expression, which may decrease miR-196a-5p to target SNX16. Proper adjustment of the BHLHE40/miR-196a-5p/SNX16 axis is able to attenuate PE symptoms.^[42]^ Nevertheless, the role of DEFA1B and FAM26D in PE remains ambiguous. Utilizing these ROC curves, we developed a diagnostic model with AUCs of 0.872 and 0.824 in the respective datasets, indicating strong performance in distinguishing between PE and healthy pregnancies. These 2 genes could potentially serve as biomarkers associated with the onset and progression of preeclampsia. However, despite investigating the roles of several hub genes identified in our study in PE, the research in this area remains insufficient.

There are some shortcomings of the present study that should be acknowledged. First of all, the validity of our conclusions mainly rests on the reliability of the original microarray dataset, which may not capture all the complexities of gene expression in PE. Secondly, our study suggests that the 7 potential genes may be diagnostic biomarkers for PE, but the role of these genes in PE is still unclear. Although we validated expressions of 4 of the selected genes, no in vivo or in vitro functional studies were carried out. Therefore, this should be a focus in future work. Finally, this study uses limited datasets from the GEO database, which may not represent the full diversity of PE cases. We would integrate more datasets and more information, such as environment, lifestyle, and so on for analysis in recent future. To our knowledge, our work is the first to use stromal and immune score combined with WGCNA to explore novel hub genes in placenta tissues associated with PE and its subgroups. Totally, robust DEGs were identified and a 7-gene risk model was constructed. The results indicated that this risk signature outperformed in predicting PE.

## 5. Conclusion

In conclusion, we have identified 7 crucial genes, including COL17A1, DEFA1B, BHLHE40, FAM26D, FSTL3, SH3BP5, and SPX based on the publicly accessible data in GEO database, which probably play vital roles in the progression of PE. Moreover, a prognostic signature based on these genes is constructed by WGCNA and LASSO regression. These findings showed that immune and stromal cells played essential roles in PE during its progression. These 7 genes are new targets for immunotherapy in PE patients. In future studies, we may be able to develop more drug sensitive therapy targeting these cells and molecules in PE microenvironment.

## Author contributions

**Conceptualization:** Hong Qin.

**Data curation:** Hong Qin.

**Formal analysis:** Hong Qin.

**Funding acquisition:** Hong Qin.

**Methodology:** Hong Qin.

**Writing – original draft:** Hong Qin.

**Writing – review & editing:** Hong Qin.

## Supplementary Material




