# Supplementary figures and images for: Detection and assessment of immune and stromal related risk genes to predict preeclampsia: A bioinformatics analysis with dataset
Source: Medicine (Baltimore). 2024 Jun 28;103(26):e38638. doi: 10.1097/MD.0000000000038638 (PMC11466178; doi:10.1097/MD.0000000000038638)

## Slide 1
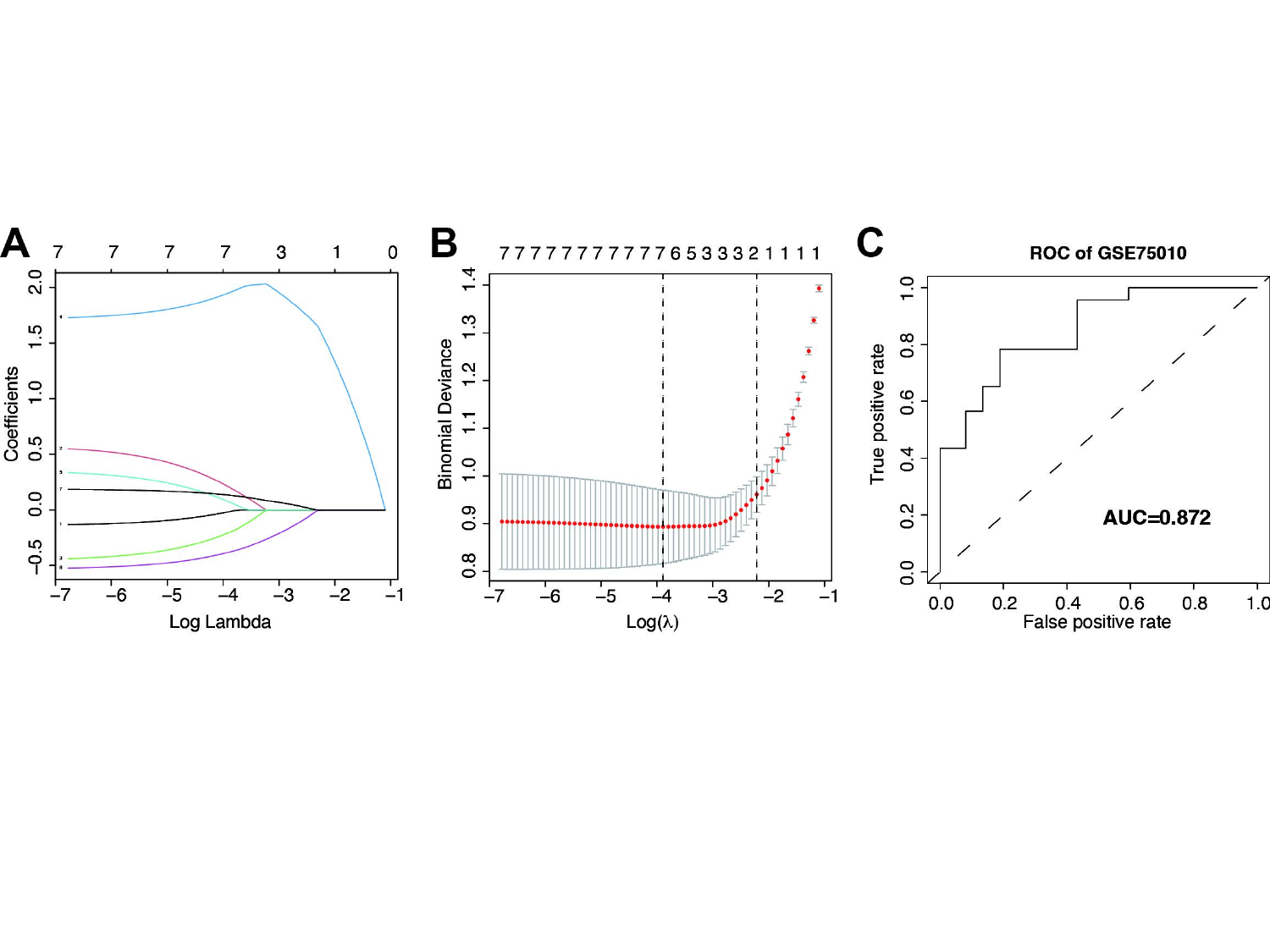

Supplement: Supplementary file 1 [file medi-103-e38638-s001.pptx]

## Slide 1
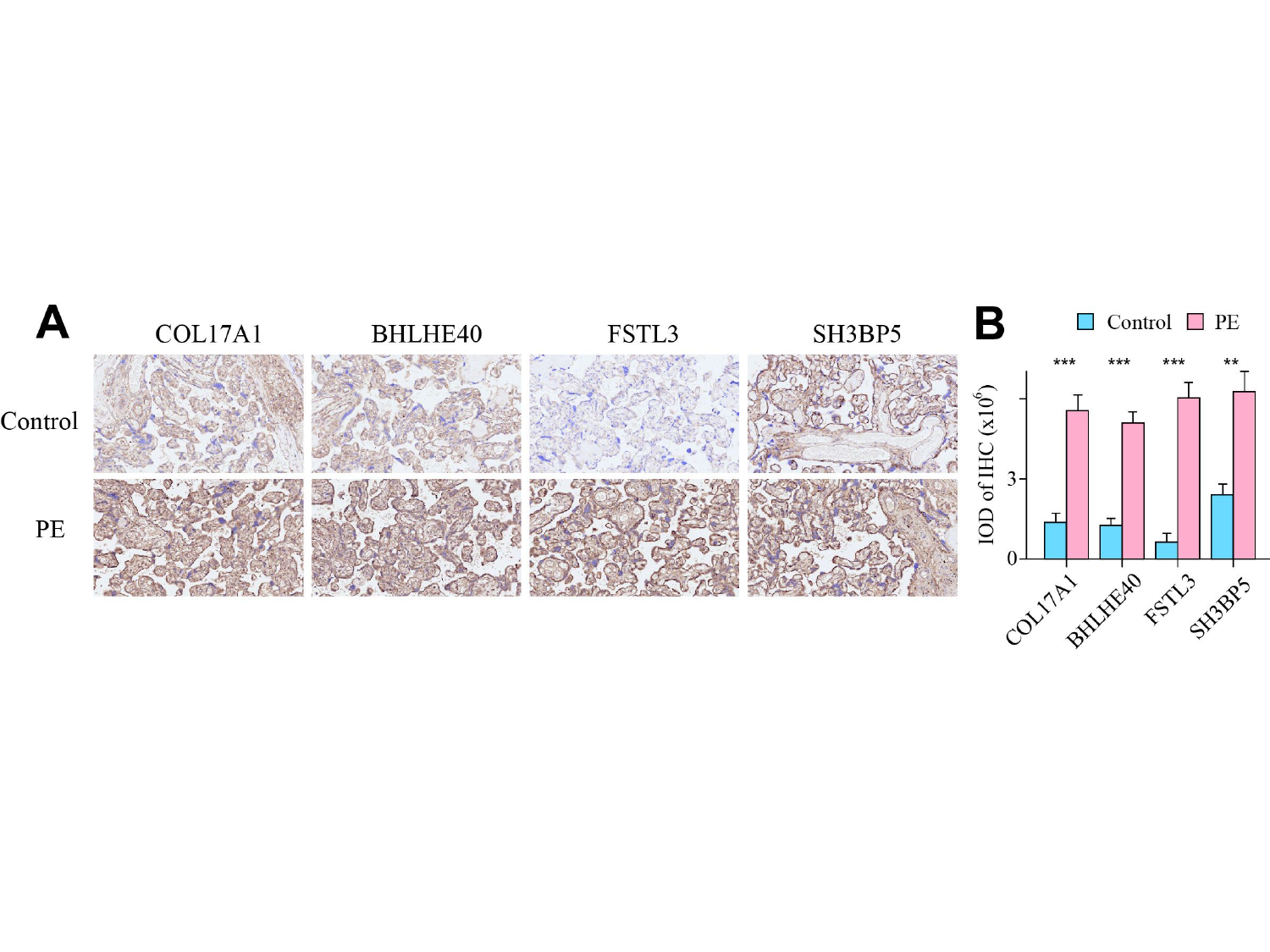

Supplement: Supplementary file 2 [file medi-103-e38638-s002.pptx]
